# Association of three missense mutations in the homocysteine-related MTHFR and MTRR gene with risk of polycystic ovary syndrome in Southern Chinese women

**DOI:** 10.1186/s12958-020-00688-8

**Published:** 2021-01-07

**Authors:** Wanqin Feng, Yan Zhang, Yuan Pan, Yi Zhang, Minjuan Liu, Yuxin Huang, Yuanling Xiao, Wenyu Mo, Junjie Jiao, Xiaoyang Wang, Dan Tian, Lixia Yang, Ying Ma

**Affiliations:** 1 Department of Gynecology, Obstetrics and Gynecology Center, Zhujiang Hospital, Southern Medical University, No.253, Gongye Middle Avenue, Haizhu District, 510280 Guangzhou, Guangdong China; 2 Clinical Research Center, Zhujiang Hospital, Southern Medical University, 510280 Guangzhou, China; 3 Department of Laboratory Medicine, Zhujiang Hospital, Southern Medical University, 510280 Guangzhou, China; 4 Department of Obstetrics and Gynecology, Dongguan People’s Hospital, 523000 Dongguan, China; 5 Department of Obstetrics, Obstetrics and Gynecology Center, Zhujiang Hospital, Southern Medical University, 510280 Guangzhou, China; 6 Department of Fetal Medicine and Prenatal Diagnosis, Obstetrics and Gynecology Center, Zhujiang Hospital, Southern Medical University, 510280 Guangzhou, China; 7 Department of Emergency, Zhujiang Hospital, Southern Medical University, 510280 Guangzhou, China

**Keywords:** Polycystic ovary syndrome, *MTHFR*, *MTRR*, Folic acid, Homocysteine

## Abstract

**Background:**

The etiology between homocysteine and polycystic ovary syndrome (PCOS) is unclear. In humans, the level of homocysteine is mainly affected by two enzymes: methylene tetrahydrofolate reductase (MTHFR) and methionine synthase reductase (MTRR). While the activity of these two enzymes is mainly affected by three missense mutations, namely C677T (MTHFR), A1298C (MTHFR), and A66G (MTRR). This study aims to examine the association between the three missense mutations and PCOS and investigate whether the three missense mutations exerted their effect on PCOS by affecting the homocysteine level.

**Methods:**

A case-control study was designed, comprising 150 people with PCOS and 300 controls. Logistic regression analysis was used to assess the association between the three missense mutations and PCOS. Linear regression analysis was used to assess the association between the three missense mutations and the homocysteine level. Mediation analysis was used to investigate whether the three missense mutations exerted their effect on PCOS by affecting the homocysteine level.

**Results:**

Following adjustments and multiple rounds of testing, *MTHFR* A1298C was found to be significantly associated with PCOS in a dose-dependent manner (compared to AA, OR = 2.142 for AC & OR = 3.755 for CC; *P* < 0.001). *MTRR* A66G was nominally associated with PCOS. Mutations in *MTHFR* A1298C and *MTRR* A66G were significantly associated with the homocysteine level. Mediation analysis suggested the effect of *MTHFR* A1298C on PCOS was mediated by homocysteine.

**Conclusions:**

*MTHFR* A1298C and *MTRR* A66G were associated with PCOS, and *MTHFR* A1298C might affect the risk of PCOS by influencing the homocysteine level.

## Background

Polycystic ovary syndrome (PCOS) is a common endocrine and metabolic disease that affects women of childbearing age. There is no cure for PCOS and it is prone to relapse [1–3]. Although the etiology of PCOS is not yet completely understood, increasing evidence suggests that it is a multifactorial disease caused by environmental and genetic factors [4–7]. Environmental factors include environmental toxins, diet and nutrition, socioeconomic status, and geography, which are believed to affect the pathogenesis of PCOS [8–10]. Moreover, mutations, polymorphisms, and differential regulation of genes may contribute to the genetic pathogenesis of PCOS [11, 12]. While a limited number of studies have reported relationships between PCOS and several candidate genes [13–15], no single gene has yet been identified as a biomarker.

Several studies have shown that an elevated homocysteine level may be related to the pathogenesis of PCOS [16–20]. A previous study found that elevated homocysteine levels could modulate M2 macrophage polarization via estrogen suppression, which promoted insulin resistance and adipose tissue inflammation in PCOS mice [20]. A multi-center, randomized, controlled trial with 936 participants showed that hyperhomocysteinemia, a medical condition characterized by an abnormally high level of homocysteine in the blood, increased the risk of pregnancy loss and reduced ovulation in people with PCOS [16].

In humans, the level of homocysteine is mainly affected by the metabolism of folic acid and methionine [21], where methylene tetrahydrofolate reductase (MTHFR) and methionine synthase reductase (MTRR) are the key enzymes [22–25]. Studies have shown that the activity of these two enzymes is mainly affected by three missense mutations, namely C677T (*MTHFR*), A1298C (*MTHFR*), and A66G (*MTRR*) [23, 26, 27]. The C677T mutation is the substitution of base C at the 677 site with T, leading to the substitution of alanine with valine, which results in a thermolabile variant with reduced activity. The mutation of *MTHFR* A1298C is the substitution of base A to C at the 1298 site, leading to the substitution of glutamic acid with alanine, which reduces enzyme activity. The *MTRR* A66G mutation alters isoleucine into a methionine residue in the protein chain and subsequently disrupts the methionine/homocysteine cycle.

Therefore, we hypothesize that C677T, A1298C, and A66G may be risk factors for PCOS, acting through an increased level of homocysteine. In this case-control study, we performed a genotype analysis for the three missense mutations to elucidate if they were risk factors for PCOS. Furthermore, we examined whether the three mutations exerted their effect in PCOS by affecting the homocysteine level.

## Materials and methods

### Participants

This study recruited 150 premenopausal women with PCOS from the Department of Gynecology and Obstetrics, Zhujiang Hospital of the Southern Medical University (Guangzhou, PR China), between December 2018 and August 2019. All cases had a definitive diagnosis of PCOS, as per the Rotterdam diagnostic criteria [28]. A total of 300 age-matched, healthy, child-bearing women without PCOS were recruited as the control group for the same period. All cases and controls had no history of cancer, diabetes, hypertension, hyperprolactinemia, Cushing’s syndrome, acromegaly, immune system disorders, a recent history of pregnancy, oral contraceptive pill for half a year prior to the study, and no previous treatment with folic acid.

Written informed consent for the study was provided by each participant. Ethical approval for the study was granted by the Institutional Research Ethics Committee of Zhujiang Hospital of Southern Medical University.

### Measures

In this study, two fasting venous blood samples were extracted from each subject. One was collected in a normal EP tube for serum measurements, and the other was collected in an anticoagulant-treated EP tube for DNA extraction. Both were stored at − 80℃ until use.

### Serum measurements

Serum homocysteine levels were quantified using a cycling enzymatic method on a Mindray BS2000M automatic biochemical analyzer (Shenzhen Mindray Bio-Medical Electronics Co., Ltd, Shenzhen, PR China). The detection limit was determined by analyzing 6 replicates of the zero calibrators and 2 replicates of the lowest nonzero calibrator. If the concentration of homocysteine was more than 50 µmol/L, the sample was manually diluted and retested. The reference range was 5–15 µmol/L.

### DNA extraction and genotyping

The DNA samples were extracted by using Magnetic Blood Genomic DNA Kit (Tiangen biochemical Technology co., Beijing, China), and were stored at -20 °C until use.

Each DNA sample was assessed for *MTRR* A66G, *MTHFR* A1298C, and *MTHFR* C677T single nucleotide polymorphisms (SNPs) using the TaqMan-MGB SNPs Genotyping Assay (Applied Bio-systems Inc., Foster City, CA, USA). Fluorescence quantitative PCR was performed for TaqMan-MGB. The reagents and primers used were all from the Human MTHFR and MTRR Gene Polymorphism Detection Kit (Fluorescence PCR; SurExam Bio-Tech Co., Ltd, Guangzhou, PR China). The PCR amplification conditions were as follows: 95 °C denaturation for 10 min followed by 20 cycles of amplification (92 °C for 15 s and 60 °C for 60 s) and 35 cycles of amplification (89 °C for 15 s and 60 °C for 90 s). All assays were replicated twice and the genotype allocation was determined by an automatic allele calling the quality value of 0.95.

### Statistical analysis

The differences in demographic and clinical characteristics were compared using the Chi-squared test or t-test. The deviation of genotype distribution was tested for Hardy–Weinberg equilibrium using the χ^2^ test. The associations between *MTHFR/MTRR* mutations and the risk of PCOS were assessed using logistic regression analyses under additive, dominant, and recessive models. Bonferroni correction was used to adjust for multiple comparisons. Linear regression was used to assess the associations between homocysteine and *MTHFR/MTRR* gene mutations. We further conducted a causal mediation analysis to test whether the association significance between *MTHFR/MTRR* mutations and PCOS was mediated via homocysteine level. Significance of the mediation effect was conducted using 5000 bootstrapped iterations mean indirect and direct effect. The bootstrapping test was performed using the SPSS PROCESS macro to test the statistical significance of the mediating effect [29].

For all multivariable models, potential confounders, including age, BMI, history of smoking, drinking, and hypertensive family history were adjusted. A two-tailed *P*-value of < 0.05 was considered statistically significant. The SPSS 19.0 statistical package was used for all data analyses.

## Results

### Participant characteristics

Table 1 shows the characteristics of the participants. The mean age of the PCOS group was 27.02 ± 4.76 years, which was not significantly different from that of the control group (*p* = 0.363). Consistent with previous meta-analysis [30], the percentage of overweight or obesity in PCOS group is higher than the control group. The PCOS group subjects had a significantly lower folate level and higher homocysteine level compared to the control subjects. For the other characteristics, there was no significant difference between participants in the two groups.

**Table 1:** Demographic and clinical characteristics of the participants

Characteristics	Control group (*n* = 300)	PCOS group (*n* = 150)	*P* value
Age (years), mean ± SD	27.44 ± 4.21	27.02 ± 4.76	0.363
Height (m), mean ± SD	1.60 ± 0.03	1.60 ± 0.04	0.994
Body mass index (Kg/m^2^), mean ± SD	20.31 ± 2.03	20.72 ± 3.01	0.131
Overweight or Obesity, n (%)	17 (5.7%)	17 (11.3%)	**0.032**
Smoking, n (%)	7 (2.3%)	5 (3.3%)	0.756
Drink, n (%)	6 (2.0%)	4 (2.7%)	0.910
Hypertensive family history, n (%)	13 (4.3%)	8 (5.3%)	0.635
Estradiol (pmol/L), median (IQR)	171.00 (135.5–218.75)	141.50 (98.75–206.25)	0.856
Prolactin (ug/L), median (IQR)	15.04 (8.52–20.58)	15.84 (10.99–25.18)	0.385
Testosterone (ug/L), median (IQR)	0.49 (0.42–0.53)	0.54 (0.44–0.68)	0.315
Follicle-stimulating hormone (IU/L, median (IQR)	8.05 (6.45–13.55)	7.05 (5.95–7.93)	0.102
Luteinizing hormone (IU/L), median (IQR)	4.54 (3.75–9.96)	5.67 (4.05–7.66)	0.554
Cholesterol (mmol/L), median (IQR)	4.66 (3.77–5.02)	4.37 (3.87–4.79)	0.157
Triglyceride (mmol/L), median (IQR)	0.84 (0.63–1.41)	0.82 (0.64–1.13)	0.561
Fasting glucose (mg/dl), median (IQR)	4.73 (4.62–5.22)	4.99 (4.81–5.24)	0.116
Fasting insulin (µU/ml), median (IQR)	5.76 (3.71–8.07)	5.24 (3.62–8.19)	0.514
Folate (ng/mL), mean ± SD	11.99 ± 2.58	10.49 ± 3.83	**< 0.001**
Homocysteine (µmol/L), mean ± SD	8.13 ± 1.21	10.07 ± 2.06	**< 0.001**

Bold values indicate significance (*P* < 0.05)

For the Hardy–Weinberg equilibrium, no significant deviation from the expected population genotype proportions was detected at the *MTHFR* C677T (χ^2^ = 2.41, *P* = 0.12), *MTHFR* A1298C (χ^2^ = 3.65, *P* = 0.06), and *MTRR* A66G (χ^2^ = 3.42, *P* = 0.06).

### Association of three missense mutations in *MTHFR* and *MTRR* genes and PCOS risk

Table 2 shows the genetic associations between the three *MTHFR/MTRR* mutations and PCOS in both univariable and multivariable additive, dominant, and recessive models. The *MTHFR* A1298C showed a significant association with PCOS under all the three models, and the association remained significant after adjusting for potential confounders and Bonferroni correction for multiple testing was applied (*P* < 0.016). In the multivariable analysis of the additive model for *MTHFR* A1298C, a clear dose dependency was observed. Compared to the reference group (AA), those who carried one risk allele (AC) had 2.142 times higher risk of developing PCOS, and those who carried two risk alleles (CC) had 3.755 times higher risk. The *MTRR* A66G was nominally associated with PCOS (0.016 < *p* < 0.05) under the additive and recessive models, but not the dominant model. There was no association between *MTHFR* C677T and PCOS.

**Table 2:** Genotype distribution of *MTHFR, MTRR* mutations in the cases and controls according to additive, dominant, and recessive models

Gene & SNP	Model	Genotype	PCOS Group	Control Group	OR (95% CI)	*P* value	Adjusted OR^a^ (95% CI)	*P* value
			N (%)	N (%)				
*MTHFR* C677T	Additive	CC	67 (44.7%)	157 (52.3%)	1	0.184	1	0.352
		CT	68 (45.3%)	109 (36.3%)	1.462 (0.964–2.217)		1.225 (0.790–1.899)	
		TT	15 (10.0%)	34 (11.3%)	1.034 (0.528–2.023)		0.753 (0.372–1.524)	
	Dominant	CT + TT	83 (55.3%)	143 (47.7%)	1	0.126	1	0.628
		CC	67 (44.7%)	157 (52.3%)	0.735 (0.496–1.090)		0.902 (0.595–1.368)	
	Recessive	TT	15 (10.0%)	34 (11.3%)	1	0.669	1	0.263
		CC + CT	135 (90%)	266 (88.7%)	1.150 (0.605–2.186)		1.467 (0.750–2.870)	
*MTHFR*A1298C	Additive	AA	65 (43.3%)	195 (65.0%)	1	**< 0.001**	1	**< 0.001**
		AC	63 (42.0%)	91 (30.3%)	2.077 (1.356–3.182)		2.142 (1.376–3.336)	
		CC	22 (14.7%)	14 (4.7%)	4.714 (2.280–9.748)		3.755 (1.741–8.096)	
	Dominant	AC + CC	85 (56.7%)	105 (35.0%)	1	**< 0.001**	1	**< 0.001**
		AA	65 (43.3%)	195 (65.0%)	0.412 (0.276–0.615)		0.422 (0.277–0.641)	
	Recessive	CC	22 (14.7%)	14 (4.7%)	1	**< 0.001**	1	**0.008**
		AA + AC	128 (85.3%)	286 (95.3%)	0.285 (0.141–0.575)		0.368 (0.176–0.771)	
*MTRR* A66G	Additive	AA	82 (54.7%)	162 (54.0%)	1	**0.014**	1	**0.028**
		AG	46 (30.7%)	118 (39.3%)	0.770 (0.500–1.186)		0.694 (0.442–1.088)	
		GG	22 (14.7%)	20 (6.7%)	2.173 (1.122–4.210)		1.796 (0.902–3.572)	
	Dominant	AG + GG	68 (45.3%)	138 (46.0%)	1	0.894	1	0.475
		AA	82 (54.7%)	162 (54.0%)	1.027 (0.693–1.523)		1.162 (0.770–1.753)	
	Recessive	GG	22 (14.7%)	20 (6.7%)	1	**0.007**	1	**0.031**
		AA + AG	128 (85.3%)	280 (93.3%)	0.416 (0.219–0.789)		0.481 (0.247–0.935)	

*OR *odds ratio, *CI *confidence intervals

^a^ Adjusted for potential confounders, including age, body mass index, folate, history of smoking, drinking, and hypertensive family history

Bold values indicate significance after Bonferroni correction for multiple comparisons (*P* < 0.016, namely 0.05/3 for three SNPs)

After removing obese women from both studied groups, we found similar results (data not shown).

### Association of three missense mutations in *MTHFR* and *MTRR* genes and serum homocysteine level

Table 3 shows associations between the three *MTHFR/MTRR* mutations and the serum homocysteine level for all participants after adjusting for potential confounders. Mutations in *MTHFR* A1298C and *MTRR* A66G were significantly associated with the serum homocysteine level. Participants having more risk alleles had a significant positive association with higher serum homocysteine levels than those with less. For example, compared to people who had no risk allele (AA), those who had one risk allele (AC) in MTHFR A1298C was 0.867 µmol/L higher in the homocysteine level, and two risk alleles (CC) was 2.092 µmol/L higher.

**Table 3:** Association of the three *MTHFR/MTRR* mutations and homocysteine levels

	Genotype	β^a^ (95% CI)	*P* value
*MTHFR* C677T	CC	reference	
	CT	0.034 (-0.124–0.193)	0.672
	TT	0.025 (-0.141–0.192)	0.764
*MTHFR* A1298C	AA	reference	
	AC	0.867 (0.577–1.158)	**< 0.001**
	CC	2.092 (1.574–2.611)	**< 0.001**
*MTRR* A66G	AA	reference	
	AG	-0.189 (-0.499–0.122)	0.233
	GG	0.704 (0.189–1.219)	**0.007**

^a^ Adjusted for potential confounders, including age, body mass index, folate, history of smoking, drinking, and hypertensive family history

Bold values indicate significance (*P* < 0.05)

### Mediation analysis

Table 4 shows whether the associations between three *MTHFR/MTRR* mutations and PCOS were mediated via homocysteine levels. The casual mediation analysis indicated that the effect of *MTHFR* A1298C on PCOS was mediated via the homocysteine level (indirect effect = 0.772 for AC; 1.861 for CC; *p* < 0.05 for both). For *MTRR* A66G, only the effect of the phenotype GG on PCOS was mediated via the homocysteine level (indirect effect = 0.623; *p* < 0.05). For both the *MTHFR* A1298C and *MTRR* A66G, no significant direct effect was observed. There was no direct or indirect association between *MTHFR* C677T and homocysteine level on PCOS.

**Table 4:** Mediation analysis of associations between three *MTHFR/MTRR* mutations and PCOS risk (mediated by homocysteine)

		Relative indirect effect (95% CI)	Relative direct effect (95% CI)	Total effect (95% CI)
*MTHFR* C677T	CC	reference	reference	-0.003 [-0.004 - (-0.002)]
	CT	0.062 (-0.240–0.354)	0.167 (-0.337–0.670)	
	TT	0.069 (-0.492–0.706)	-0.431 (-1.260–0.399)	
*MTHFR* A1298C	AA	reference	reference	**0.102 (0.052–0.168)**
	AC	**0.772 (0.464–1.132)**	0.155 (-0.353–0.663)	
	CC	**1.861 (1.085–2.777)**	-0.054 (-1.092–0.985)	
*MTRR* A66G	AA	reference	reference	0.014 (-0.001–0.036)
	AG	-0.169 (-0.473–0.115)	-0.344 (-0.855–0.166)	
	GG	**0.630 (0.149–1.211)**	0.135 (-0.693–0.963)	

All estimates obtained was adjusted for potential confounders, including age, body mass index, folate, history of smoking, drinking, and hypertensive family history, and used causal mediation analysis with SPSS’s Process command

Relative Direct effects: independent effects of three missense mutations on PCOS risk. Relative Indirect effects: effects of three missense mutations on PCOS risk (mediated by homocysteine)

Bold values indicate significance (*P* < 0.05)

## Discussion

Studies showed that an elevated homocysteine level may be related to the pathogenesis of PCOS. In this case-control study, we found that two mutations in homocysteine-related genes, namely *MTHFR* A1298C and *MTRR* A66G, were associated with the risk of PCOS, and the associations were mediated through influencing the level of homocysteine.

In our study, *MTHFR* A1298C was highly associated with PCOS after adjusting for potential confounders. Compared to the wild-type genotype AA, mutant homozygote genotype AC had a 2.142 times higher risk of PCOS, and CC had 3.755 times higher risk of PCOS, which showed that the effect size was stronger for each additional risk allele C. In agreement with our study, a recent meta-analysis demonstrated that *MTHFR* A1298C was associated with PCOS susceptibility [31]. For *MTRR* A66G, our results suggest an association with PCOS, but no significant association was found after multiple tests. A case-control study with 203 Brazilian participants showed that the polymorphic homozygous mutation of *MTRR* A66G was associated with protective factors for PCOS [32]. For the *MTHFR* C677T, we did not observe an association with PCOS; conflicting results have been reported, ranging from no observed association Poland [33] to the T allele being a risk factor for PCOS in the Korean population [34]. Such different conclusions of studies may be due to genetic mutations, race, region, and other factors, or due to differences in sample size.

Importantly, we also found that the association between *MTHFR* A1298C and PCOS was mediated by the serum homocysteine level. Mediation analysis examines what proportion of the SNP-PCOS association travels through homocysteine and acknowledges the fraction of the association that is independent of homocysteine. The result of the mediation analysis showed that the effect of A1298C on PCOS was through homocysteine. To our knowledge, this is the first study examining the potential mechanism underlying the association between genetic factors and the risk of PCOS. In addition, there is a biologically expected direction for the effect of A1298C on homocysteine and PCOS, in that A1298C with more risk alleles increased homocysteine levels and the risk of PCOS, which supports the mediation analysis results.

Homocysteine is a protein that is synthesized in the body and, ideally, is in low concentrations in the blood [35]. Elevated homocysteine is a risk factor for many diseases, including PCOS and cardiovascular disease [36–41]. Therefore, our finding of the association between the homocysteine-related mutations and PCOS supports previous studies. However, to confirm the causality of the homocysteine levels and PCOS, a mendelian randomization study is needed. As the mutations of A1298C affect the function of MTHFR, drugs that treat MTHFR may reduce the risk of PCOS. Furthermore, as people with PCOS have a higher risk of cardiovascular disease, there may be an assumption that PCOS patients with a *MTHFR* A1298C AC/CC genotype may be prone to cardiovascular disease [42]. A key strength of this study is that we used mediation analysis to explain the biological rationality of the association between the risk mutations and PCOS. Furthermore, potential confounders were taken into account in the regression analyses.

However, several study limitations exist. Similar to other case-control studies, our study has limited verification of causality, but with the observations of other epidemiological supports from the Bradford-Hill Criteria [43], such as the temporal relation between the genes and PCOS outcome, the strong magnitude of effect, clear allele dose dependency, and biological plausibility, our findings increased the possibility of causality. Although the percentage of overweight or obesity in PCOS group is significantly higher than the control group (11.3% vs. 5.7%), there might be some inclusion bias since the pooled estimated prevalence of overweight and obesity in people with PCOS was 61% [30]. Another limitation is that we used convenience samples to examine the relationship between the risk alleles and homocysteine level, which prevented control of the representativeness of the samples. This lack of control may cause biased samples and research results, and thus limit the wider application of the study. In addition, as the association between the three mutations with PCOS may be ethnic-specific and the interactions between genes and the environment may also modulate PCOS risk, the findings of the present study need to be verified in different populations and other larger cohort studies.

In conclusion, *MTHFR* A1298C and *MTRR* A66G were associated with PCOS, and *MTHFR* A1298C might affect the risk of PCOS by influencing the homocysteine level. Drugs that treat MTHFR may reduce the risk of PCOS for people with the *MTHFR* A1298C AC/CC genotype.

## Data Availability

The datasets used and analysed during the current study are available from the corresponding author on reasonable request.

## Abbreviations

PCOS: Polycystic ovary syndrome
MTHFR: Methylene tetrahydrofolate reductase
MTRR: Methionine synthase reductase
SNPs: Single nucleotide polymorphisms
